# High Performance Liquid Chromatography Analysis and Description of Purine Content of Diets Suitable for Dogs with *Leishmania* Infection during Allopurinol Treatment—A Pilot Trial

**DOI:** 10.3390/ani13193060

**Published:** 2023-09-29

**Authors:** Melanie Kaempfle, Michèle Bergmann, Petra Koelle, Katrin Hartmann

**Affiliations:** LMU Small Animal Clinic, Centre for Clinical Veterinary Medicine, LMU Munich, 80539 Munich, Germany; n.bergmann@medizinische-kleintierklinik.de (M.B.); p.koelle@medizinische-kleintierklinik.de (P.K.); hartmann@lmu.de (K.H.)

**Keywords:** leishmaniosis, low purine, canine, dog diet, urolithiasis, xanthine, xanthinuria, urinary stones

## Abstract

**Simple Summary:**

Allopurinol is commonly used for the treatment of canine *Leishmania* infections. In order to reduce the risk of xanthine urolith formation, a considerable adverse effect of allopurinol, a low purine diet is mandatory. Several dog diets are advertised as low in purine, but standardised analyses are missing. Thus, the present study analysed the purine content of such dog diets (4 purine urolith prevention diets, 2 kidney diets, 1 vegan diet, 3 low protein diets, 1 homemade diet) and 1 regular diet by high performance liquid chromatography. The results revealed differences in the purine content of the diets. The lowest purine intakes could be achieved by 2 of the urate urolith prevention diets and the homemade diet. In conclusion, awareness has to be raised when selecting diets for dogs with *Leishmania* infections during allopurinol treatment.

**Abstract:**

Reducing the alimentary purine intake contributes to the prevention of purine (especially xanthine) urolith formation, a common adverse effect of allopurinol treatment in dogs with *Leishmania* infections. Analyses of the purine content are not required in order to advertise a diet as low in purine. Due to different analytical methods, data provided on purine content are barely comparable. The aim of this study was to investigate the total purine content of 12 different dog diets. For this, the purine bases adenine, guanine, xanthine, and hypoxanthine were determined by standardised high performance liquid chromatography in commercially available urinary diets (n = 4), kidney diets (n = 2), low protein diets (n = 3), 1 vegan diet, 1 regular diet for healthy adult dogs, and 1 homemade low purine diet. Total purine amounts ranged between 10.2 and 90.9 mg/100 g of dry matter. The daily purine intake calculated for a 20 kg standard dog with the analysed diets ranged between 21.9 and 174.7 mg. The lowest daily purine intakes were achieved by 2 urinary urate diets, followed by the homemade diet. Differences in the purine content of commercially available diets need to be considered. Awareness has to be raised when selecting diets for dogs with *Leishmania* infections during allopurinol treatment in order to minimise the risk of urolith formation.

## 1. Introduction

In endemic areas in Europe (e.g., Italy, France, Spain, Portugal), approximately 2.5 million dogs are infected with *Leishmania* spp., the causing agent of the parasitic infectious disease canine leishmaniosis [1]. Transmission of these protozoa and infection of dogs mainly occurs through the bite of infected female sandfly vectors of the genus *Phlebotomus* in Europe. It is assumed that the perimeter of the natural geographic distribution of the vector is related to the annual mean temperature isotherm of 10 °C and correlates with the endemic distribution of canine leishmaniosis. The northern boundary of the endemic area in Europe is marked by the foothills of the Alps and Pyrenees but currently spreads further north due to climate changes [2,3]. In non-endemic areas, infections with *Leishmania* spp. occur mainly in dogs that were imported from or have visited endemic areas [4]. However, individual cases of autochthonous transmission have also been observed [5,6]. While some infected dogs develop the clinical disease that can manifest in lesions of different organs, including the skin, others never develop signs of canine leishmaniosis [7,8,9,10].

For the treatment of infected dogs, different drugs are available. The purine analogue allopurinol, alone or in combination with meglumine antimoniate or miltefosine, is commonly used as the gold standard basic treatment [11]. The therapeutic effect of allopurinol is based on the inability of *Leishmania* to synthesise purines de novo [12]. Purines are N-heterocycles (C_5_H_4_N_4_) widespread in nature, where they occur in substituted form only [13]. Purine derivatives can be classified into oxypurines (xanthine, hypoxanthine, uric acid, allantoin), aminopurines (guanine, adenine), and methylpurines (caffeine, theophylline, theobromine) [14]. The purine bases adenine and guanine are components of DNA and RNA. Furthermore, purine derivatives (especially adenosine triphosphate and adenosine) are essential for energy metabolism and serve as transmitters [15]. *Leishmania* require purines provided by their hosts. During allopurinol treatment, the parasites metabolise allopurinol and integrate it into their RNA, which effectively inhibits *Leishmania* replication [16,17,18,19].

**Figure 1:**
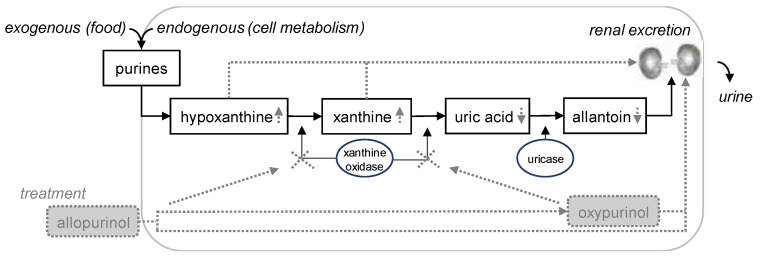
Effect of allopurinol treatment on urinary excretion of purine catabolites in dogs. Endogenous (originating from cell metabolism) and exogenous (originating from food intake) purines are catabolised to hypoxanthine and xanthine. In healthy dogs without allopurinol treatment (solid black lines), the enzyme xanthine oxidase catalyses the oxidation of hypoxanthine to xanthine and xanthine to uric acid. Uric acid is oxidised to allantoin, catalysed by the enzyme uricase. Allantoin is well soluble in water and excreted in urine through the kidneys. In the case of treatment with allopurinol (dotted grey lines), that is a xanthine oxidase inhibitor similar to its metabolite oxypurinol, large amounts of the poorly water-soluble xanthine and hypoxanthine are excreted in urine through the kidneys and thus increase the risk of purine urolith formation [24,25].

Dogs are able to synthesise purines de novo and ingest purines with food [20]. Treatment with allopurinol affects the degradation of purines in dogs [21,22]. Purines are catabolised via several purine intermediates to uric acid and finally to allantoin, a catabolite well soluble in and excreted by urine. By inhibition of the enzyme xanthine oxidase, allopurinol and its metabolite oxypurinol cause increased accumulation of the purine intermediates hypoxanthine and xanthine. Poorly soluble in water, xanthine can precipitate in the dog’s urine in case of oversaturation [14,23] (Figure 1). Most animal and plant-based food products and feed materials contain purines (Table 1).

The total purine amount in food is determined by the amount of adenine, guanine, xanthine, and hypoxanthine, the 4 purine bases that can be catabolised to uric acid. In food, these 4 purine bases are rarely free but mainly contained in DNA and RNA or in the form of nucleotides and nucleosides in low molecular compounds [30]. Thus, food products with high amounts of nucleic acid contain high amounts of purines. The amount of alimentary intake of purines correlates with the excretion rate of purines. Thus, in terms of optimal feeding regimes for dogs infected with *Leishmania* spp. and receiving allopurinol treatment, a low purine dog diet is an important issue for veterinarians, caregivers, and the pet food industry [21]. The aim is to restrict the amount of accumulating xanthine in the urine of dogs treated with allopurinol and thus to lower the risk of xanthinuria and the formation of xanthine uroliths, the most common allopurinol adverse effects [14,31,32,33,34,35,36].

To meet low purine feeding requirements, several commercially available urolith prevention diets are advertised as suitable for dogs with leishmaniosis. Values for maximum purine amounts in dog diets do not exist in the official feed law of the European Union (Commission Regulation (EU) 2020/354 of 4 March 2020). For manufacturers, it is not mandatory to declare (or even analyse) the purine content of dog diets. If declared, values are not necessarily comparable due to potentially different methods of analysis. High performance liquid chromatography (HPLC) is a standardised analytical method for the determination of the amount of the 4 purine bases adenine, guanine, xanthine, and hypoxanthine in food products [28,30,37,38].

The purpose of this study was to analyse the purine content of 12 dog diets (11 commercially available dog diets and 1 homemade diet) using a standardised HPLC method in order to find the most appropriate way of keeping the alimentary purine intake as low as possible in dogs with *Leishmania* infection treated with allopurinol.

## 2. Materials and Methods

### 2.1. Dog Diets

A total of 12 dog diets were analysed in the years 2019 and 2020, including 11 dog diets considered to be low in purine (10/11 were commercially available dog diets, 1/11 was a homemade dog diet) and 1 regular diet for adult dogs for comparison (Table 2).

Among the commercially available dog diets, the following had certain dietetic indications as declared by the manufacturers: 3 dry urinary diets intended for the prevention of urinary urate stones (urinary diet 1–3), 1 dry urinary diet specifically advertised for the prevention of urinary xanthine stones during allopurinol treatment in dogs with *Leishmania* infection (urinary diet 4), and 2 dry kidney diets intended to support the renal function in case of chronic or acute kidney failure (kidney diet 1 and 2). All the diets with special dietetic indications were from the veterinary product lines of 4 different manufacturers. Furthermore, 1dry vegan dog diet advertised as suitable for dogs with leishmaniosis by a manufacturer specialised in vegan dog diets, and 3 varieties of a canned low protein dog diet (low protein dog diet 1–3) from the veterinarian product line of a local manufacturer, were analysed. Product names and manufacturers can be requested from the corresponding author. Additionally, 1 homemade diet was analysed, which fully covered nutritional requirements and was composed regarding the criterion “low purine” for dogs undergoing allopurinol treatment, designed by the pet nutrition consultation service of the LMU Small Animal Clinic, Munich, Germany. The homemade diet consisted of different protein sources (curd, egg, and low fat beef muscle meat for better acceptance), boiled potatoes, carrots, linseed oil, and a mineral and vitamin supplement for homemade diets (Vitamin Optimix Cooking^®^, Futtermedicus, Fürstenfeldbruck, Germany). The regular (non low purine) dog diet that was included for comparison reasons was aimed to cover the daily requirements of healthy, medium-sized (11–25 kg body weight) adult dogs (ages 1 to 7 years) and was not advertised as low in purine content.

### 2.2. Purine Base Measurement and Calculations

The analysis of the purine base content of all dog diets by HPLC was carried out at the Chemical Investigation Office at the Department of Chemistry, Universität Hamburg, Germany. Freeze-dried sample equivalents of 0.1 g were weighed into a Teflon^®^ pressure digestion vessel, mixed with 10 mL formic acid/trifluoroacetic acid (1:1; *v*/*v*) and decomposed at 235 °C. The acid was removed in vacuo, and the residue was taken up to 10 mL in the elution buffer, membrane filtered, and analysed by HPLC using an Agilent 1220 UV system (Agilent Technologies, Inc., Santa Clara, CA, USA).

The separation of the nucleobases was achieved on a LiChroCart^®^ LiChrospher^®^ (Merck, Düsseldorf, Germany) 100 RP-18 end-capped (250 × 4 mm) column with a LiChroCart^®^ LiChrospher^®^ (Merck, Düsseldorf, Germany) 100 RP-18 end-capped (4 × 4 mm) precolumn under isocratic conditions. The eluent consisted of 0.02 mol ammonium dihydrogen phosphate and 5 mmol dimethyl octyl amine (pH 3.10), the flow rate was 1 mL/min, and the injection volume was 20 μL using a sample loop. The detection of the analytes by the ultraviolet (UV) detector was achieved at 254 nm. The data system used was the Open-LAB ChemStation (Agilent Technologies, Inc., Santa Clara, CA, USA). The amounts of the 4 purine bases, adenine, guanine, hypoxanthine, and xanthine were determined by external standards and standard addition in the hydrolysates.

For the calculation of the amount of total purine nitrogen contained in the wet weight of the dog diets, the analysed amount of each purine base was multiplied with a base-specific conversion factor (the ratio of the molecular weight of the 4 nitrogen atoms contained in the purine elementary structure and the molecular weight of the respective base), and the results were added [38]. The uric acid equivalent, referred to as the wet weight, a value that specifies the amount of uric acid metabolised out of the contained purines in humans, was calculated by multiplying the total purine nitrogen by a factor of 3. Afterwards, the purine nitrogen content and uric acid equivalent were additionally calculated related to the dry matters of the dog diets.

The results were applied to the daily food intake of an exemplary 20 kg standard dog, assuming a daily maintenance energy requirement of 898 kcal, based on the estimated values for pet dogs from the National Research Council (MER = 95 kcal × kg BW^0.75^) [39,40].

For the calculation of the daily amount of each diet necessary to cover the standard dog’s energy requirements, the energy density of each analysed diet was calculated by Diet Check Munich^™^ (RV Software, Unterschleißheim, Germany). This calculator system comprises data on components of homemade diets based on digestion trials and analyses [26,41]. If data on digestibility are not available (e.g., in prepared diets), the metabolisable energy (ME) was calculated using a predictive calculation recommended for prepared diets [39,41,42].

In a final step, the resulting daily purine intake associated with the required daily food intake of the standard 20 kg dog was calculated. The calculations were performed with the software program Microsoft Excel, version 2108 (Microsoft Corporation, Redmond, Washington, DC, USA).

## 3. Results

The results of the HPLC analysis revealed differences in the purine base profile of all dog diets (Table 3). In the analysed dog diets, adenine values ranged between 3.2 and 54.5 mg/100 g of the wet weight, guanine content between 4.3 and 52.4 mg/100 g, hypoxanthine between <0.1 and 27.4 mg/100 g, and xanthine between <0.1 and 7.2 mg/100 g. The analysed vegan dog diet and all diets with certain dietetic indications (except urinary diet 4) contained very low amounts of hypoxanthine and xanthine; the purine base content was mainly composed of adenine and guanine.

The further calculation of total purine nitrogen and uric acid equivalents revealed a similar pattern. Purine nitrogen content ranged between 5.0 and 42.1 mg/100 g of the wet weight, with resulting values of uric acid equivalents between 14.9 and 126.3 mg/100 g. The purine nitrogen content of the dry matter ranged between 10.2 and 90.9 mg/100 g, with resulting values of uric acid equivalents between 30.5 and 272.6 mg/100 g. The estimated energy density of the dry dog diets ranged between 354 and 406 kcal per 100 g wet weight, and the energy density of the canned diet and the homemade diet ranged between 97 and 118 kcal/100 g of the wet weight.

Thus, the daily amount of a diet that an exemplary standard dog of 20 kg body weight would need to cover his daily maintenance energy requirement ranged between 221.0 and 926.0 g. The associated amount of purine intake ranged between 21.9 and 174.7 mg/day. The lowest daily purine intake of 21.9 mg was found in urinary diet 1. The second lowest daily purine intake of 25.2 mg was detected in urinary diet 2. The third lowest daily purine intake of 28.5 mg was contained in the homemade diet. The highest daily purine intakes were calculated for the vegan dog diet, followed by 2 of the low protein dog diets (low protein dog diets 2 and 3), with the highest daily purine intake of 174.7 mg, which was approximately 8 times higher than the lowest calculated daily purine intake (urinary diet 1: 21.9 mg/day).

## 4. Discussion

The present study analysed the purine content of 12 different dog diets with a standardised HPLC method for a low purine feeding regime in dogs with *Leishmania* infections undergoing allopurinol treatment. The mean daily purine intake of a 20 kg standard dog with a diet advertised or considered to be low in purine was 75.1 mg (range: 21.9–174.7 mg). In 5 of these diets, the calculated purine intake exceeded the purine intake associated with the regular dog diet that was included in the study for comparison reasons (82.7 mg). The lowest daily purine intake calculated for a 20 kg standard dog was associated with urinary diets 1 and 2. Both diets are designed by market-leading companies, and veterinarians are involved in the development of the formulations. Apart from recommendations for allopurinol treatment in *Leishmania* infections, a low purine diet is indicated in dogs with congenital disorders of purine metabolism, e.g., the genetic predisposition to the formation of urate uroliths in Dalmatian dogs [14,35,43]. Diets for the prevention of such urate stones aim to keep the purine content low and can therefore be suitable for dogs with *Leishmania* infections undergoing allopurinol treatment as well. Since the Dalmatian is frequently discussed as a model for hyperuricemia and gout in humans, nutritional studies are mainly conducted with this dog breed and data about non-Dalmatian dogs with *Leishmania* infections and allopurinol treatment are rare. In a previous study on 6 Dalmatian dogs in 2003, for example, urinary diet 2 of the present study was compared to 4 different conventional non-urinary dog diets and an all-meat dog diet regarding the amount of formed urinary precipitates. Quantification of urinary precipitates was performed visually. With highly significant differences among the investigated dog diets, dogs that received urinary diet 2 or one other of the conventional dog diets produced the lowest amounts of precipitates in the urine [44].

However, in urinary diet 2, potential problems of low protein content, especially in long-term feeding, are often discussed. In the past, a series of idiopathic cardiomyopathies in 9 Dalmatian dogs was described, assuming that feeding the low protein and low purine urinary diet 2 could possibly have contributed to the diseases. Of the investigated Dalmatians, 8 out of the 9 dogs were fed with urinary diet 2 for a part or all of their lives, with a mean feeding time of 33.4 months (6–72 months). Since an insufficient carnitine supply could not be ruled out, it was suspected that the disease was related to that diet [45]. Notably, the protein content referring to the dry matter of urinary diet 2 (10.8%) is lower than an amount of at least 18% protein in the dry matter, which is recommended by the Association of American Feed Control Officials (AAFCO). Nevertheless, this diet was approved by AAFCO maintenance feeding trials and thus can serve as an adequate diet for adult dogs [14]. Only for dogs in growth or reproduction, having higher protein requirements, a low protein dog diet should be avoided [14]. Generally, it has to be considered that the evaluation of food protein content should not only take the protein quantity into account but also the respective biological value and the amino acid composition, which can vary significantly depending on the protein source used and possible amino acid complementation by the manufacturers.

Urinary diet 1, however, had a higher protein content than urinary diet 2, which, with 19.6% of the dry matter, is in line with the AAFCO minimum recommendations. In a study in mainly Dalmatian dogs fed with urinary diet 1 over a period of 12 months, lower excretion values of urinary uric acid compared to baseline values were achieved by the diet, accompanied by a high acceptance [35].

The daily purine intake calculated for a 20 kg standard dog with a homemade low purine, low fat diet was 28.5 mg. A possibility to further reduce the total daily purine intake of this ration could be achieved by adding fat to the ration, which is, however, not a possible practice for every dog, e.g., in case of obesity, pancreatitis or intestinal health issues.

The daily purine intakes of the 2 commercial kidney diets analysed were 33.4 mg (kidney diet 2) and 40.2 mg (kidney diet 1). In order to protect the kidneys (from further disease), such diets focus on a low protein and phosphorus content [46,47]. However, dog diets low in protein are not necessarily low in purine [28]. The choice of an adequate source of protein plays an important role in terms of purine content. Since purines are catabolites of DNA and RNA, the amount of purines in a specific protein source is related to the amount of containing cell nuclei. For example, high amounts of purines are contained in offal, meat extracts (e.g. bouillon), salmon, tuna, and yeast. Due to their relatively high purine content, lentils and other legumes, such as (soy)beans, should be avoided in a low purine feeding regime [14]. In conclusion, focusing on a low purine feeding regime, kidney diets seem less suitable than other diets.

Urinary diets 3 and 4 were associated with daily purine intakes between 62.5 and 83.9 mg. Interestingly, urinary diet 4 is specifically marketed as a diet for dogs with *Leishmania* infections during allopurinol treatment and, therefore, widely recommended by veterinarians for this indication, which, in fact, is very dangerous (at least in the evaluated batch of this diet). It has to be mentioned, however, that the manufacturer of urinary diet 4 claimed that the diet had been modified in the meantime. Further analyses might therefore be interesting.

The highest daily purine intakes were calculated for the 3 varieties of the low protein dog diet (between 92.7 and 174.7 mg) and the vegan dog diet (106.9 mg). As cell-rich lentils were chosen as a protein source for the vegan dog diet, it is not extremely surprising that it had a relatively high daily purine intake of 106.9 mg. Vegan dog diets do not contain any ingredients of animal origin. Thus, the choice of protein source is limited and purine-rich legumes, such as peas or lentils, are inevitably frequently used to cover the dog’s daily protein requirements. In contrast, many vegetarian protein sources, such as eggs, consisting of a single cell nucleus, are high in protein but low in purines. Together with whole grain and milk products (for example), they are suitable protein sources in purine-restricted dog diets. However, commercially available vegetarian dog diets (that might be believed to be low in purine, e.g., by dogs’ caregivers) often contain purine-rich additives, such as yeast cells or seaweed, to ensure an adequate iodine supply and thus are not automatically a good choice for low purine feeding management.

In the prevention of hyperuricemia and gout in human medicine, it is known that not only the total amount of purines ingested is important but also the profile of the various purine bases, especially the amount of consumed hypoxanthine [28,48]. Previous research on this topic indicates that, e.g., purine-rich vegetables, with a purine content composed mainly of adenine and guanine, do not affect uric acid plasma levels when consumed moderately, which is different in an equal total amount of purine, mainly composed of hypoxanthine, contained in high amounts in most animal meat, including fish [49]. For dogs, data on this topic is rare [20]. Especially regarding the vegan dog diet with a high total purine amount but a purine base profile mainly consisting of adenine and guanine, it would be interesting to know whether the amount of individual purine bases is important in pet nutrition, too. However, the sole use of vegan dog diets in general remains to be discussed. A recently published study evaluating vegan diets available in Germany for dogs and cats demonstrated that none of the analysed dog diets fully met the recommendations for energy and nutrient supply for adult dogs [50].

What could be misleading is the claim by some manufacturers that a diet is specifically suitable for dogs with leishmaniosis (such as in the urinary diet 4). Due to the high adaptability of *Leishmania* to purine restriction, there is no evidence of a benefit of low purine diets for infected dogs that are not treated with allopurinol, assuming that the parasites are able to cover their purine requirements only with the dogs’ endogenously synthesised purines [51,52]. Thus, the purine amount of a diet is not relevant in dogs with leishmaniosis without allopurinol treatment. However, allopurinol is applied to almost every dog with leishmaniosis as this is the gold standard basic treatment.

Apart from dietary restrictions for dogs undergoing allopurinol treatment, other advices to lower the risk for purine stone formation should include forced upregulation of water intake and urine alkalisation, thereby preventing an oversaturation and precipitation of xanthine in the urine [14,34]. The dissolution of xanthine mineralisation by influencing the urine pH is not possible, but urine alkalisation can help in the prevention of new precipitates. Since the urine pH is mainly influenced by food intake and different dog diet additives with urine alkalinising effect (e.g., kalium citrate), regular urine analysis, including pH measurement, should be conducted to monitor the conditions when dogs receive a specific diet. Feeding can also partly contribute to a good fluid intake [53]. Nevertheless, some of the dog diets analysed are available only as dry dog diets, such as urinary diet 1. A possible explanation for being only available as a dry dog diet is the usually higher meat content in canned diets, resulting in an even higher total purine amount [44]. Consequently, care should be taken to ensure good additional water intake when feeding dry diets only, for example, by soaking in water before feeding. To provide data on other feeding options, further studies about the purine content, even for varieties that are also available as canned diets, such as urinary diet 2, should be conducted in the future.

The limitations of the present study are that the exact composition of a dog diet is subject to natural variations, and different batches of the same diet can differ slightly. Depending on the availability of individual ingredients on the world market and the source of supply, there are frequently minor changes in the formulation of every dog diet. The results of the present study refer to the versions of the individual dog diets available on the German market between 2019 and 2020. To provide data on variations of the purine content of each diet itself, further studies with repeated analyses of different batches of the diets and determination of statistically significant differences are necessary. Additionally, it has to be mentioned that the purine content of the diet does not allow direct conclusions on the xanthine concentration in the urine and the resulting risk for xanthine urolithiasis since it is further influenced by (amongst others) urine concentration and pH, allopurinol dose, and type of purines consumed [31,36]. Nevertheless, as shown in a previous study, feeding dogs with a diet considered low in purine (canned version of urinary diet 2; supplemented with potassium citrate) led to significantly lower urine concentrations of xanthine and uric acid in comparison to a meat-based diet [31].

So far, studies ont the prevention of urinary purine stone formation with low purine diets have mainly been conducted in healthy dogs and Dalmatians [20,32,35,43,44,45,54,55,56]. Further studies about the effectiveness of such diets, with special regard to the purine base profile of the diets and their importance, should also be conducted in dogs infected with *Leishmania* and receiving allopurinol treatment.

## 5. Conclusions

Standardised HPLC analysis provides the possibility of measuring the purine content in dog diets. Based on the results of this study, 2 commercially available dog diets intended for the reduction of urinary urate stones followed by 1 homemade, low purine diet were associated with the lowest daily purine intake calculated for a 20 kg standard dog. Since xanthinuria and xanthine stone formation during allopurinol treatment of dogs with *Leishmania* infections are the most common adverse effects, caregivers need to be adequately and thoroughly instructed on all measures of xanthine urolith prevention, especially a low purine feeding regimen. For early detection, regular checks, including ultrasound examinations of the urinary tract and urinalyses, are essential.

## Figures and Tables

**Table 1:** Examples of feed materials with high, medium, and low purine content [26].

Feed Material		DM (g)	Total Purines (mg Uric Acid)	
Category	Example	/100 g WW	/100 g WW	/100 g DM
*High purine content*				
**Yeast**	Baker yeast	27.0	680	2519
	Liver (pork)	28.0	515	1839
**Organs**	Kidney (beef)	23.9	269	1126
	Heart (beef)	24.5	256	1045
	Sardines	24.0	345	1438
**Fish**	Salmon	34.5	170	493
	Tuna	38.5	179	465
**Seaweed**	Nori (dry)	89 *	696 **	782
*Medium purine content*				
	Chicken (muscle)	30.6	115	376
**Meat**	Beef (brisket)	33.6	90	268
	Pork (belly)	39.7	100	252
**Legumes**	French beans	10.5	37	352
	Pea (pod and seed)	24.8	84	339
	Soybean (dry seed)	91.6	190	207
	Processed soybeans (tofu)	15.4	68	442
**Edible insects**	Mealworm (*Tenebrio molitor*)	32.9 ***	112	339 ***
	Cricket (*Acheta domesticus*)	30.2 ***	82	270 ***
*Low purine content*				
**Vegetable**	Carrot	11.8	17	144
	Potato	22.2	16	72
**Grain**	Wheat (whole grain)	87.3	51	58
**Dairy**	Yogurt, 3.5% fat	13.0	8	62
	Cottage cheese	21.5	9	44
**Egg**	Chicken	25.3	0 **	0

DM, dry matter; WW, wet weight; * Cofrades et al., 2008 [27]; ** Kaneko et al., 2014 [28]; *** Sabolová et al., 2023 [29].

**Table 2:** Dog diets enrolled in the study (available in Germany in the years 2019 and 2020), including texture, dry matter, crude protein (in wet weight and in dry matter), and manufacturer declaration of indication and the main protein sources.

Dog Diet *	Texture	DM ^1^ (%)	CP (% WW)	CP (% DM)	Manufacturer * Declaration	
					Indication	Main Protein Sources
**Urinary dog diets**						
U1	dry	91.5	18	20	Prevention of cystine and urate uroliths	Dried egg, wheat gluten feed, corn gluten feed
U2	dry	92.5	10	11	Reduction of urate and cystine urolith formation, Reduction of copper storage in the liver in adult animals	Dried whole egg
U3	dry	91.0	23	25	Reduction of formation of oxalic, urate and cystine stones	Potato protein, flaked peas, dried whole egg
U4	dry	94.0	24	26	Prevention of xanthine uroliths specifically for dogs with *Leishmania* infection treated with allopurinol	Dehydrated egg, soy protein, hydrolysed animal protein, plasma proteins, caseinate
**Kidney dog diets**						
K1	dry	91.5	14	15	Support of renal function in case of chronic renal insufficiency, reduction of ingredient and nutrient intolerances	Soy protein isolate
K2	dry	92.5	14	15	Support renal function in chronic or acute renal insufficiency in adult dogs	Dried whole egg, pea protein
**Vegan dog diet**						
V	dry	91.3	24	26	No specifically advertised indication	Lentils, peas
**Low protein dog diets**						
Lp1	canned	22.7	6	26	No specifically advertised indication	Wild game muscle meat, chia seeds
Lp2	canned	21.2	6	28	No specifically advertised indication	Calf heart, calf lung, chia seeds
Lp3	canned	22.4	6	27	No specifically advertised indication	Chicken muscle meat, chicken stomach, chicken skin, chia seeds
**Homemade low purine dog diet**						
Hm	cooked	24.5	8	33	Prevention of xanthine uroliths specifically for dogs with *Leishmania* infection treated with allopurinol	Curd, egg, beef muscle meat
**Regular dog diet**						
R	dry	91.5	23	25	No specifically advertised indication	Dried poultry protein, hydrolysed animal protein, dried pig protein, wheat feed flour, wheat gluten, corn, rice, beet chips

DM, dry matter; CP, crude protein; WW, wet weight; ^1^ requested from manufacturer if not declared; U1–3, urinary dog diets intended for the prevention of urinary urate stones; U4, urinary dog diet specifically advertised for the prevention of urinary xanthine stones during allopurinol treatment in dogs with *Leishmania* infections; K1–2, kidney dog diets for support of renal function in case of acute or chronic kidney failure; V, vegan dog diet advertised as suitable for dogs with leishmaniosis; LP1-3, low protein dog diets; HM, homemade low purine dog diet composed by the pet nutrition consultation service of the LMU Small Animal Clinic, Munich, Germany; R, regular diet for adult dogs, not advertised as a low purine dog diet. * Product names and manufacturers can be requested from the corresponding author.

**Table 3:** Results of purine base analysis of different dog diets by high performance liquid chromatography and calculation of the purine amount, uric acid equivalent, energy density, and daily purine intake calculated for a dog of 20 kg body weight.

HPLC Analyses					Calculated Values							
Dog Diet *	Purine Bases				Purine N		Uric Acid Equivalent		Energy	Purine N	Daily Intake	
	mg/100 g WW				mg/100 g		mg/100 g		kcal/100 g	mg/1000 kcal	g	mg
	A	G	HX	X	WW	DM	WW	DM	WW		WW	Purine N
**Urinary dog diets**												
U1	9.8	14.1	<0.01	<0.01	9.3	10.2	27.9	30.5	383	24	235.0	**21.9**
U2	11.5	17.2	<0.1	<0.1	11.2	12.1	33.6	36.3	399	28	225.0	**25.2**
U3	29.9	35.5	2.2	<0.1	26.5	29.1	79.5	87.4	380	70	236.0	62.5
U4	34.4	41.5	10.8	4.1	35.7	38.0	107.0	113.8	382	93	235.0	83.9
**Kidney dog diets**												
K1	16.8	29.2	<0.1	<0.1	17.8	19.5	53.4	58.4	398	45	226.0	40.2
K2	15.3	23.7	<0.1	<0.1	15.1	16.3	45.3	49.0	406	37	221.0	33.4
**Vegan dog diet**												
V	54.5	52.4	<0.1	<0.1	42.1	46.1	126.3	138.3	354	119	254.0	106.9
**Low protein dog diets**												
LP1	4.5	6.8	18.9	<0.1	12.2	53.7	36.5	160.8	118	103	761.0	92.7
LP2	12.1	12.7	16.8	7.2	19.3	90.9	57.8	272.6	111	173	809.0	155.8
LP3	12.3	13.9	20.8	<0.1	18.9	84.2	56.6	252.7	97	194	926.0	174.7
**Homemade low purine dog diet**												
Hm	3.2	4.3	5.0	<0.1	5.0	20.2	14.9	60.7	114	43	575.0	**28.5**
**Regular dog diet**												
R	23.8	35.4	27.4	3.3	35.5	38.8	106.5	116.4	386	92	233.0	82.7

HPLC, high performance liquid chromatography; DM, dry matter; WW, wet weight; A, adenine; G, guanine; HX, hypoxanthine; X, xanthine; purine N, amount of nitrogen bound in the purine ring of the purine bases adenine, guanine, xanthine and hypoxanthine; U1–3, urinary dog diets intended for the prevention of urinary urate stones; U4, urinary dog diet specifically advertised for the prevention of urinary xanthine stones during allopurinol treatment in dogs with *Leishmania* infections; K1–2, kidney dog diets for support of renal function in case of acute or chronic kidney failure; V, vegan dog diet advertised as suitable for dogs with leishmaniosis; LP1-3, low protein dog diets; HM, homemade low purine diet composed by the pet nutrition consultation service of the LMU Small Animal Clinic, Munich, Germany; R, regular dog diet for adult dogs, not advertised as low purine dog diet; bold numbers: dog diet with daily purine intake less than 30 mg. * Product names and manufacturers can be requested from the corresponding author.

## Data Availability

The data presented in this study are available on request from the corresponding author.
